# 

**Published:** 1886-08

**Authors:** 


					﻿AUGUST, 1886
<-QLD SERIES^
Vol. XXV
, & 1855
NEW SERIES
Vol. III.-No. 6.*
WmP
HEDK^wSlii
111 IK |\j |v ■Wfc/’
X eJIb iM J-I | I	a

b w.f.westmoreTand. i® j
H.VM.MILLER.M.DJ.L .D.®t
JAMES A.GRAY. MU.*’
V0UBLISHED KY JAMES RHARR1SON&CQS|
Me	Atlanta, ga. J&S
SUBSCRIPTIONS 12.50 A YEAR.
				

